# Bibliometric and visual analysis of blood-testis barrier research

**DOI:** 10.3389/fphar.2022.969257

**Published:** 2022-08-22

**Authors:** Yifeng Shen, Yaodong You, Kun Zhu, Chunyan Fang, Xujun Yu, Degui Chang

**Affiliations:** ^1^ TCM Regulating Metabolic Diseases Key Laboratory of Sichuan Province, Hospital of Chengdu University of Traditional Chinese Medicine, Chengdu, China; ^2^ School of Medicine and Life Sciences, Chengdu University of Traditional Chinese Medicine, Chengdu, China

**Keywords:** blood-testis barrier (BTB), male infertility, citespace, vosviewer, bibliometrics

## Abstract

**Background:** Extensive research on the blood-testis barrier has been undertaken in recent years. However, no systematic bibliometric study has been conducted on this subject. Our research aimed to identify the hotspots and frontiers of blood-testis barrier research and to serve as a guide for future scientific research and decision-making in the field.

**Methods:** Studies on the blood-testis barrier were found in the Web of Science Core Collection. VOSviewer, CiteSpace, and Microsoft Excel were used to conduct the bibliometric and visual analyses.

**Results:** We found 942 blood-testis barrier studies published in English between 1992 and 2022. The number of annual publications and citations increased significantly between 2011 and 2022, notably in the United States. China and the United States, the US Population Council, Endocrinology, and Cheng C. Yan were the most productive countries, institution, journal, and author, respectively. The study keywords indicated that blood-testis barrier research involves a variety of compositional features (tight junctions, cytoskeleton, adherens junctions), cell types (Sertoli cells, germ cells, Leydig cells, stem cells), reproductive toxicity (cadmium, nanoparticles, bisphenol-a), and relevant mechanisms (spermatogenesis, apoptosis, oxidative stress, dynamics, inflammation, immune privilege).

**Conclusion:** The composition and molecular processes of the blood-testis barrier as well as the blood-testis barrier in male infertility patients are the primary research hotspots in this field. In addition, future research will likely focus on treatment and the development of novel medications that target signal pathways in oxidative stress and apoptosis to preserve the blood-testis barrier. Further studies must extend to clinical diagnosis and therapy.

## Introduction

Blood-tissue barriers prevent large molecules from being exchanged uncontrollably across metabolically distinct compartments (Alves et al., 2012). In the mammalian testis, the blood-testis barrier (BTB) is a unique ultrastructure. The BTB comprises coexisting tight junctions (TJs), basal ectoplasmic specialization (basal ES), desmosomes and gap junctions between neighboring Sertoli cells beneath the seminiferous tubule’s basement membrane; this feature is unlike that of other blood-tissue barriers, such as the blood-brain barrier and the blood-ocular (or blood-retina) barrier, created by TJs between endothelial cells of microvessels. The BTB separates the seminiferous epithelium into apical (or adluminal) and basal compartments, allowing meiosis I and II as well as post-meiotic germ cell development to occur in a specific microenvironment behind the BTB (Cheng et al., 2011). The BTB prevents the innate immune system from recognizing haploid germ cells. Furthermore, when germ cells are translocated into the adluminal compartment, their nutritional supply is isolated from the circulatory system, and they are fed solely by Sertoli cells.

Male infertility is hypothesized to be linked to the BTB. Thirty to forty percent of males with aberrant semen parameters have unexplained infertility. Infertile individuals’ spermatogenic abnormalities are caused by a variety of factors. The most common cause of unexplained male infertility is endocrine disturbance of testicular development during the neonatal period due to environmental pollutants and genetic and epigenetic factors (Ghafouri-Fard et al., 2021). These variables have been linked to testicular dysgenesis, infertility, and testicular cancer. These multiple factors are thought to be linked to the BTB and may have a role in the etiopathology of male infertility by disrupting BTB regulation (Jiang et al., 2014). Continuous research into BTB mechanisms has aided in the discovery of the best treatment to enhance patient prognosis. Until recently, no effective medicine for treating male infertility associated with the BTB has been authorized. Drugs that ameliorate BTB injury induced by cadmium in the rat testis have been shown in animal studies to reduce BTB damage and may be potential drugs for male infertility treatment. For example, melatonin ameliorates BTB injury induced by cadmium in the rat testis (Venditti et al., 2021), and vitamins E and C reduce di-(2-ethylhexyl) phthalate-induced BTB disruption in rats (Shen et al., 2018). Treatments based on molecular processes, cellular signaling networks, and gene control offer tremendous therapeutic promise. However, a viable therapeutic method based on the BTB has yet to be established.

Bibliometric analysis, which concentrates on the systems and characteristics of the literature, is frequently used to comprehend the knowledge structure and discover emerging trends using qualitative and quantitative study of the scientific literature. The contributions of different authors, institutions, nations, and publications can be compared, and a certain study subject can be summarized, and the future growth of that field can be projected. Developing guidelines, comprehending research hotspots, and assessing research trends all rely heavily on this analytical technique. Health research (Wang et al., 2020), environmental protection (Chen et al., 2022), and reproductive medicine (Deng et al., 2022) have been studied using this method.

BTB research has progressed in the 30 years since it was initially conceived, but there are still certain challenges to be resolved. How to determine the function and structure of the BTB in patients, how to employ effective medications to treat male infertility caused by BTB damage, and whether BTB-related target drugs are being developed remain to be resolved.

In this study, we used the Web of Science Core Collection (WoSCC) to carry out a bibliometric analysis and visually assess the research hotspots of the BTB field. The results revealed the global research trends in this field, which might lead to new design ideas for future study and illuminate fundamental and clinical investigations.

## Materials and methods

### Sources of data and search strategies

On 31 May 2020, the linked publications from the start of the databases until 2022 were collected from the Science Citation Index Expanded (SCI-E) core database via the WoSCC. With the search term “blood-testis barrier” and the literature categories “article” and “review,” a total of 942 items were found that met the search parameters and were analyzed further.

### Data collection and analysis

The authors worked independently to gather and screen the WoSCC raw data. The data were analyzed using VOSviewer, CiteSpace, and Excel. Dissension was resolved through dialog. We investigated journal associations, evaluated collaborative teams across nations, institutions, and writers, created visualization maps, captured keywords, and discovered cocited authors/references using CiteSpace IV and VOSviewer.

## Results

### Publications

**FIGURE 1:**
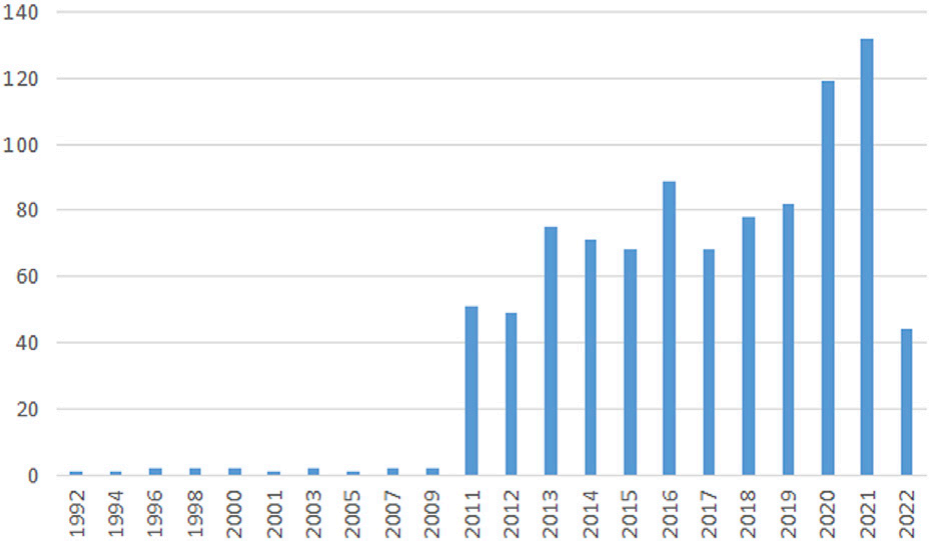
Publication output. The annual number of publications on blood-testis barrier research from 1992 to 2022.

There were a total of 942 articles that met the retrieval criteria. Figure 1 depicts the total number of articles published each year, with the trend ranging from one paper in 1992 to 132 papers in 2021. Since 2011, there has been a significant increase in the number of research papers published. The average number of articles published every year was 32, and the number of publications per year was consistent. In 2022, 44 articles had been published as of 31 May 2020.

### Countries and institutes

**FIGURE 2:**
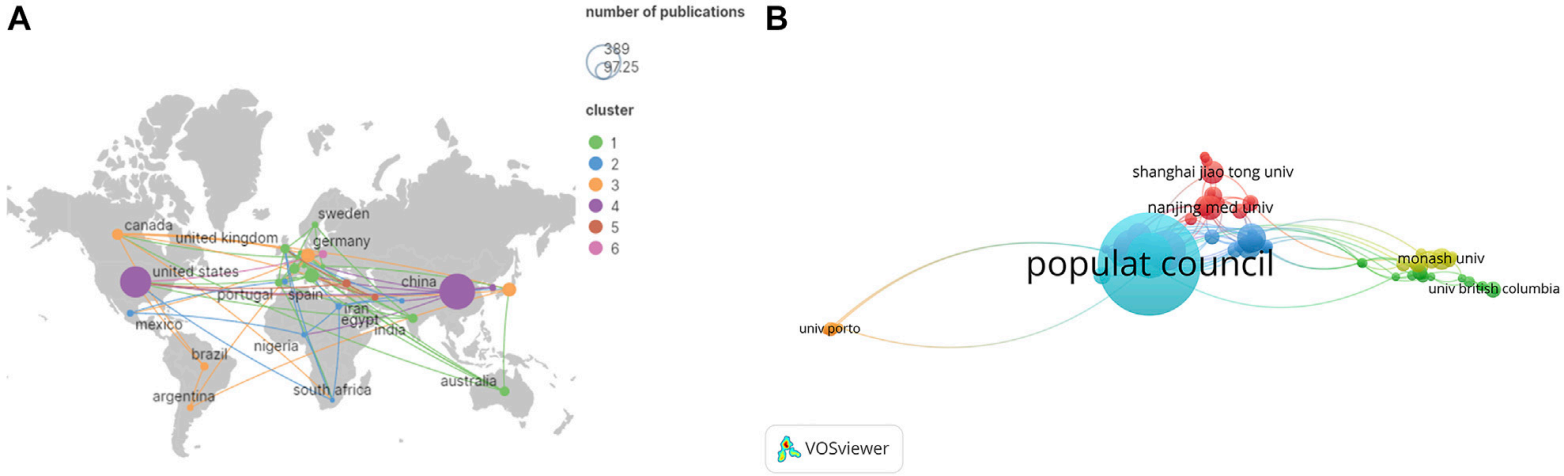
The distribution of countries and institutes. **(A)** The network map of countries/regions involved in blood-testis barrier research. **(B)** The network map of institutes involved in blood-testis barrier research.

A total of 57 regions/countries have published studies on the BTB. These regions/countries had a diverse range of collaborative teams (Figure 2A). China contributed 389 articles among the top ten regions/countries (Table 1) active in BTB research, followed by the United States (294), Italy (62), Japan (58), and Germany (57). According to the findings, over 1,058 institutes have conducted research on the BTB. A number of collaborations among institutes were identified (Figure 2B). The top ten institutes were responsible for almost 36% of all publications (Table 1). The ranking was headed by the US Population Council, which was followed by the Univ Hong Kong, Wenzhou Medical Univ, Hong Kong Baptist Univ, and Chinese Acad Sci.

**TABLE 1:** The top 20 countries and institutes publications in blood-testis barrier research.

Country/region	Count	Percent (%)	Institute	Count	Percent (%)
China	389	41.30	Populat Council	130	13.80
United States	294	31.21	Univ Hong Kong	44	4.67
Italy	62	6.58	Wenzhou Med Univ	37	3.93
Japan	58	6.16	Hong Kong Baptist Univ	34	3.61
Germany	57	6.05	Chinese Acad Sci	24	2.55
Canada	38	4.03	Huazhong Univ Sci and Technol	19	2.02
France	32	3.40	Nanjing Med Univ	19	2.02
Australia	29	3.08	Nantong Univ	19	2.02
India	27	2.87	Shanghai Jiao Tong Univ	18	1.91
United Kingdom	26	2.76	Monash Univ	16	1.70

### Journal and subject

**FIGURE 3:**
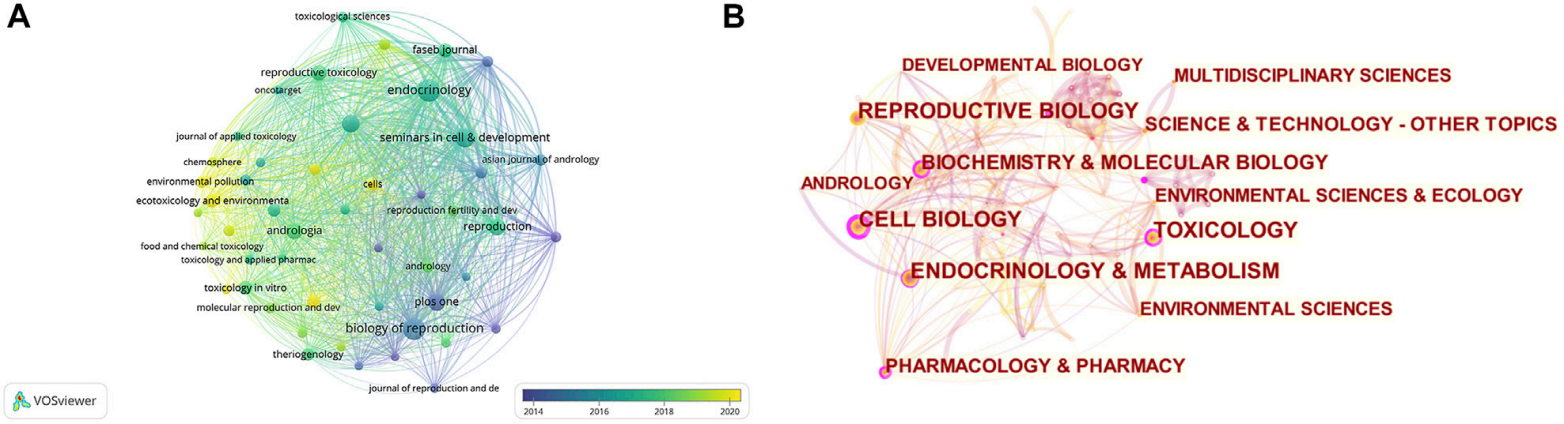
The distribution of journals and subjects. **(A)** The distribution of journals. **(B)** The distribution of subjects.

The BTB papers from 1992 to 2022 were predominantly disseminated in various journals, according to the data analysis, and the top 10 journals are summarized in Table 2; Figure 3. With 32 publications, Endocrinology was the most productive journal. The impact factors of the various journals publishing BTB research in 2021 varied from 3.4 to 12.9, with Seminars in Cell and Developmental Biology having the greatest impact factor and Andrologia having the lowest. According to the Journal Citation Reports (JCR) partition analysis, Q1 was 70% and Q2 was 30% in this ranking. Reproductive and developmental biology, endocrinology and metabolism, biochemistry and cellular molecular biology, pharmacology, environment and ecology, toxicology, and andrology were among the topics covered in the literature.

**TABLE 2:** Top 10 with the largest number of publications.

Journals	Documents	2021 impact factor	2021 JCR partition
Endocrinology	32	8.0	Q1
Biology Of Reproduction	31	5.6	Q1
Seminars In Cell and Developmental Biology	23	12.9	Q1
Scientific Reports	20	7.1	Q1
Plos One	19	5.3	Q1
Andrologia	18	3.4	Q2
Reproduction	18	6.0	Q1
Reproductive Toxicology	16	5.3	Q2
Faseb Journal	13	6.1	Q2
Ecotoxicology And Environmental Safety	12	8.2	Q1

### Authors

**FIGURE 4:**
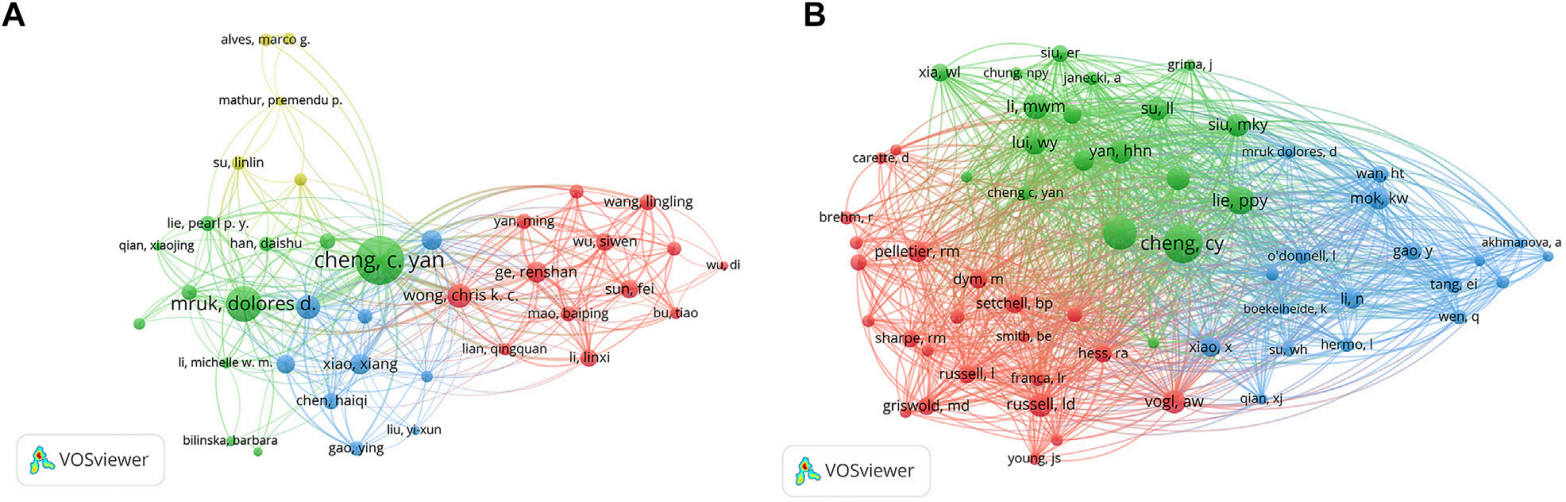
The distribution of authors. **(A)** Collaboration network among the authors. **(B)** Cocitation network among the authors.

A total of 4,300 authors were involved in BTB research. The authors’ collaborative network is depicted in Figure 4A. Cheng C. Yan (133, AUTHORID publications) was placed first among the top 10 contributing authors (Table 3), followed by Mruk, Dolores D. (73 publications), Lee, Will M. (33 publications), Wong, Chris K. C. (33 publications), Ge, Renshan (24 publications), and Xiao, Xiang (24 publications). In a cocitation network, the data of author citations were studied (Figure 4B). Cheng C. Yan (805 cocitations) was placed first among the top 10 cocited authors (Table 3), followed by Mruk, DD (597 cocitations), Lie, PPY (416 cocitations), Li, MWM (335 cocitations), and Yan, HHN (294 cocitations).

**TABLE 3:** The top 10 authors, co-cited authors in blood-testis barrier research.

Rank	Author	Count	Co-cited author	Count
1	Cheng, C. Yan	133	Cheng, CY	805
2	Mruk, Dolores D	73	Mruk, DD	597
3	Lee, Will M	33	Lie, PPY	416
4	Wong, Chris K. C	33	Li, MWM	335
5	Ge, Renshan	24	Yan, HHN	294
6	Xiao, Xiang	24	Russell, LD	292
7	Silvestrini, Bruno	23	Lui, WY	281
8	Lui, Wing-Yee	20	Vogl, AW	271
9	Sun, Fei	19	Wong, EWP	271
10	Li, Linxi	17	Siu, MKY	270

### Citations

**FIGURE 5:**
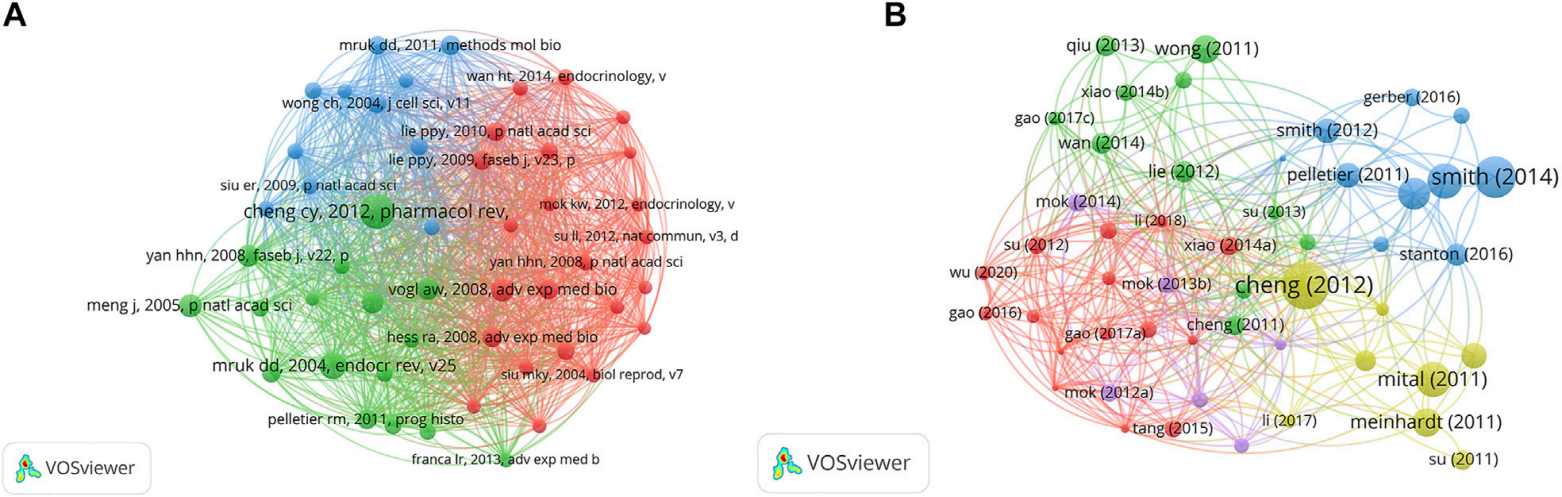
The distribution of citations. **(A)** Citation analysis of documents. **(B)** Cocitation analysis of documents.

The top 10 papers with the most citations are presented in Table 4 according to the citation analysis of the documents (Figure 5A); the range of citations was from 111 to 430. “The blood-testis barrier and its implications for male contraception” (2012), published by Cheng C. Yan, had 430 citations, followed by “The regulation of spermatogenesis by androgens”, published by Smith LB (2014), which had 324 citations, and the third was “The Mammalian Blood-Testis Barrier: Its Biology and Regulation” (2015) with 228 citations. To analyze the citations of the documents, cocitation analysis of the cited references was performed (Figure 5B; Table 5). There were a total of 40,575 cited references; the most highly cited references were Cheng C. Yan (Cheng and Mruk, 2012), Mruk DD (Mruk and Cheng, 2004), Vogl AW (Vogl et al., 2008), Meng J (Meng et al., 2005), Cheng C. Yan (Cheng and Mruk, 2002), Cheng C. Yan (Cheng and Mruk, 2010), Yan HHN (Yan et al., 2008), Mazk DD (Yan et al., 2008), O’donnell Liza (O'Donnell et al., 2011), and Pelletier RM (Pelletier, 2011).

**TABLE 4:** | Top 10 citation analysis of documents on blood-testis barrier.

Rank	Title	First author	Source	Publication year	Total citations
1	The blood-testis barrier and its implications for male contraception	Cheng CY	Pharmacol Rev	2012	430
2	The regulation of spermatogenesis by androgens	Smith LB	Semin Cell Dev Biol	2014	324
3	The Mammalian Blood-Testis Barrier: Its Biology and Regulation	Mruk DD	Endocr Rev	2015	228
4	The blood-testis and blood-epididymis barriers are more than just their tight junctions	Mital P	Biol Reprod	2011	225
5	The Sertoli cell: one hundred 50 years of beauty and plasticity	França LR	Andrology	2016	184
6	Immunological, paracrine and endocrine aspects of testicular immune privilege	Meinhardt A	Mol Cell Endocrinol	2011	152
7	Impacts of environmental toxicants on male reproductive dysfunction	Wong EW	Trends Pharmacol Sci	2011	142
8	Sertoli cells--immunological sentinels of spermatogenesis	Kaur G	Semin Cell Dev Biol	2014	119
9	Germ cell migration across Sertoli cell tight junctions	Smith BE	Science	2012	115
10	The blood-testis barrier: the junctional permeability, the proteins and the lipids	Pelletier RM	Prog Histochem Cytochem	2011	111

**TABLE 5:** | Top 10 cocitation analysis of documents on blood-testis barrier.

Rank	Title	First author	Source	Publication year	Total citations
1	The blood-testis barrier and its implications for male contraception	Cheng CY	Pharmacol Rev	2012	275
2	Sertoli-Sertoli and Sertoli-germ cell interactions and their significance in germ cell movement in the seminiferous epithelium during spermatogenesis	Mruk DD	Endocr Rev	2004	159
3	The Sertoli cell cytoskeleton	Vogl AW	Adv Exp Med Biol	2008	116
4	Androgens regulate the permeability of the blood-testis barrier	Meng J	Proc Natl Acad Sci United States	2005	115
5	Cell junction dynamics in the testis: Sertoli-germ cell interactions and male contraceptive development	Cheng CY	Physiol Rev	2002	106
6	A local autocrine axis in the testes that regulates spermatogenesis	Cheng CY	Nat Rev Endocrinol	2010	103
7	Blood-testis barrier dynamics are regulated by testosterone and cytokines via their differential effects on the kinetics of protein endocytosis and recycling in Sertoli cells	Yan HHN	FASEB J	2008	102
8	An *in vitro* system to study Sertoli cell blood-testis barrier dynamics	Mruk DD	Methods Mol Biol	2011	84
9	Spermiation: the process of sperm release	O’donnell Liza	Spermatogenesis	2011	84
10	The blood-testis barrier: the junctional permeability, the proteins and the lipids	Pelletier RM	Prog Histochem Cytochem	2011	83

### Keywords

**FIGURE 6:**
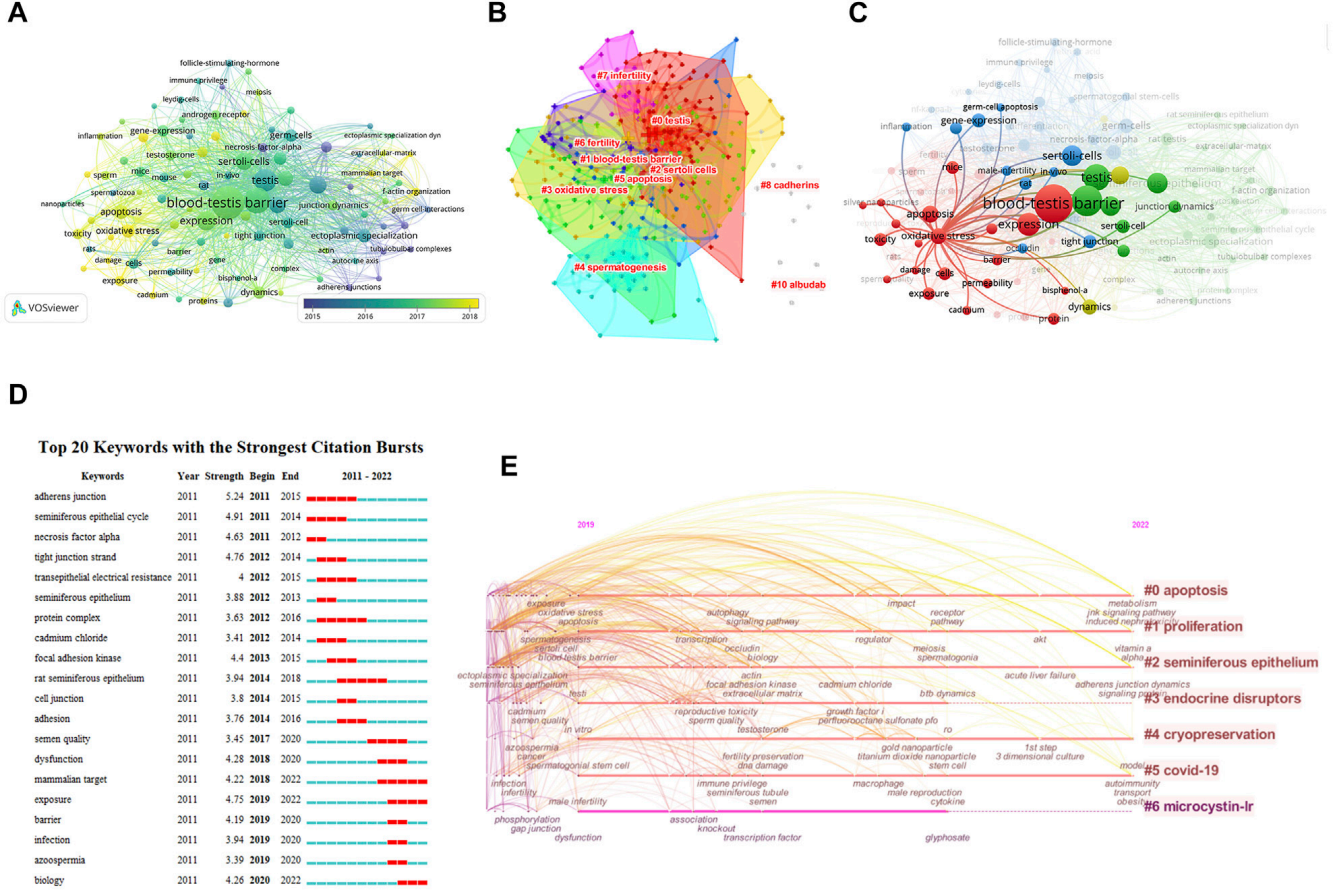
Co-occurrence analysis of keywords. **(A)** The analysis method was Linlog/modularity in VOSviewer, the weight was an occurrence, and scores were the average published year. The thickness of the lines indicates the strength of the relationship. The color indicates the average publication year. **(B)** Keyword cluster map. **(C)** The relationship between oxidative stress and apoptosis and other keywords was examined by network visualization. **(D)** Burst word. **(E)** Keyword timeline for the past 3 years.

There were 2,853 keywords in all; 292 keywords were presented in a minimum of five documents. The colors in Figure 6A overlay depiction represent the average publication year of the discovered keywords. The majority of the keywords, with greener or yellower hues, were used after 2015. Blood-testis barrier, spermatogenesis, seminiferous epithelium, Sertoli cells, apoptosis, ectoplasmic specialization, oxidative stress, germ cells, dynamics, and tight junctions were high-frequency keywords. The keywords could be divided into the following categories: BTB composition, BTB-related structure and cells, male infertility and reproductive poison, and relevant mechanisms (Figure 6B). According to statistical analysis of the keywords, numerous molecules participate in the progression of BTB composition and dynamics regulation related to male infertility and other cell types and are mainly involved in the processes of apoptosis and oxidative stress (Figure 6C). CiteSpace was utilized to analyze terms with a high citation explosion (Figure 6D): adherens junction (2011–2015), seminiferous epithelial cycle (2011–2014), necrosis factor alpha (2011–2012), tight junction strand (2012-2014), transepithelial electrical resistance (2012-2015), seminiferous epithelium (2012-2013), protein complex (2012-2016), cadmium chloride (2012-2014), focal adhesion kinase (2013-2015), cell junction (2014-2015), adhesion (2014-2016), dysfunction (2018-2020), exposure (2019-2022), infection (2019-2020), and azoospermia (2019-2020). A keyword timeline showing research trends over the past 3 years revolves around the following categories (Figure 6E): apoptosis, proliferation, seminiferous epithelium, endocrine disruptors, cryopreservation, COVID-19, and microcystin-LR.

## Discussion

### General trends

We used bibliometric analysis and network visualization to characterize the current landscape of BTB research, analyzing the contributions of countries, institutions, journals, and authors to this emerging field and predicting hot topics that will continue to be of research interest in the coming years. Since the field’s inception in 1992, yearly publishing output has consistently increased, with particularly significant growth over the last 12 years, accounting for 98 percent of all papers identified. China and the United States are now the world leaders in BTB research, with the most publications and citations as well as the highest ranking for coauthorship analysis by nation. These findings show that China and the US may have a considerable effect on the direction of research in this subject and that their collaboration is among the strongest in the world. Over the last decade, the number of studies undertaken in Italy, Japan, and Germany has also increased significantly. The most prolific institution, the US Population Council, was placed at the top in coauthorship analyses undertaken by the institution, indicating strong collaboration with other institutions.

### Influential authors and studies

Cheng C. Yan of the US Population Council is a pioneer in the field of BTB research, with the most publications and citations as well as the highest h-index, m-index, and coauthorship analysis. Cheng C. Yan is an internationally known male reproductive biologist/reproductive endocrinologist who has made major contributions to the study of testicular Sertoli cell-spermatogenic cell interactions, Sertoli cell-Sertoli cell interconnections, and BTB processes (Cheng and Mruk, 2011; Mok et al., 2013a; Mok et al., 2014). He pioneered the development of an *in vitro* compound culture model of the interaction between Sertoli cells and spermatogenic cells as well as research into the molecular mechanisms of spermatogenesis, which is now extensively utilized throughout the world (Mruk and Cheng, 2011). He has also been involved in research into the impact of environmental toxins on reproductive function, the role of drug transporters in male contraception, and the structure and dynamics of the BTB (Su et al., 2011; Wong and Cheng, 2011; Cheng and Mruk, 2012; Mruk and Cheng, 2015). Dolores D. Mruk and Cheng C. Yan, both from the US Population Council, are members of the same study team. Cheng C. Yan et al. (Pharmacol Rev, 2012) revealed that a “new” BTB is produced below spermatocytes in transit, while the “old” BTB above transiting cells undergoes timely degeneration, such that the immunological barrier may be maintained as spermatocytes traverse the BTB, which offers several potential targets for revolutionary contraceptive development and research (Cheng and Mruk, 2012). Their prior research analyzed the biology and control of the mammalian BTB (Mruk and Cheng, 2015) and offered a perspective of the testis, which includes the ectoplasmic specialization and tubulobulbar complex as well as the regulatory molecules that “open” and “close” connections concerning germ cell migration (Mruk and Cheng, 2004). Lee B Smith (Smith and Walker, 2014) of the University of Edinburgh reported that the BTB is made up of anatomical, physiological, and immunological barriers. The anatomical barrier that prevents molecules and cells from entering or departing the lumen is formed by TJs. The physiological barrier is made up of transporters that control the flow of chemicals into and out of the lumen, providing a microenvironment that is essential for germ cell growth and maturation. The immunological barrier prevents the immune system from reaching the bulk of autoantigenic germ cells and sequesters them.

### Future outlook

The current hot topics and future prospects in BTB research are highlighted in our co-occurrence network maps, which are clustered by topic area or publication date (Figure 6). The following are the most recent topics that suggest future developments in this sector.

### Role of the blood-testis barrier in male fertility

Male infertility is becoming more common, and sperm quality is deteriorating, which has been linked to rapid industrialization and the release of an abundance of synthetic compounds into the environment. Humans are invariably exposed to these ubiquitously dispersed environmental pollutants, which have the capacity to interfere with the growth and function of male reproductive organs (Selvaraju et al., 2021). The BTB is a main target of a variety of environmental toxins. Endocrine-disrupting chemicals (EDCs) are thought to induce testicular damage by interfering with the activity of steroid hormones. Alternatively, EDCs may exert their effects by interacting with ATP-binding cassette (ABC) transporters found in the BTB (Dankers et al., 2013). The endocrine disruptor bisphenol A (BPA) (Pena-Corona et al., 2021) and its structural analogs bisphenol S (BPS) (Wu et al., 2021), bisphenol AF (BPAF) (Wu et al., 2019), and tetrabromobisphenol A (TBBPA) (Dankers et al., 2013) are increasingly being utilized in consumer items and have been found to be reproductive toxins and to induce BTB damage in animal models. The indiscriminate use of heavy metals in several sectors, including industrial, agricultural, health care, cosmetics, and household sectors, has polluted environmental matrices and constitutes a serious health risk. Cadmium has been shown to inhibit systemic immunity and damage the testicular capillary endothelia, resulting in testicular tissue necrosis in mice and rats given large dosages. It affects the integrity of the BTB, the endocrine function of Leydig cells, germ cell apoptosis, and systemic immunity, even when taken at a low level that does not cause spermatogenic disruption (Ogawa et al., 2013; Marettova et al., 2015). Per- and polyfluoroalkyl substances (PFASs) are a class of synthetic compounds found in a wide range of goods, including water and oil repellents, lubricants, and firefighting foam (Steves et al., 2018). Perfluorooctanesulfonate (PFOS) perturbs the Sertoli cell TJ-permeability barrier, which disrupts actin microfilaments in the cell cytoplasm, allowing cell junction proteins to be misplaced. Sertoli cell BTB integrity is destabilized by these modifications (Li et al., 2016). Methamidophos (MET) is an organophosphate (OP) insecticide commonly used to grow crops in developing nations. This insecticide disrupts spermatogenesis by disrupting the BTB and causing immature germ cells to emerge in the epididymis (Ortega-Olvera et al., 2018). PM2.5 accumulates in the reproductive organs via the BTB, which can disturb the BTB and hormone levels, resulting in infertility (Wang et al., 2021). Ionizing radiation causes male infertility and increases the permeability of the BTB (Son et al., 2015). Nanoproducts are becoming more frequently employed in many sectors of life as the development of nanometer-sized materials accelerates. Nanoparticles (NPs) may cause structural damage to the BTB as well as an increase in irritability in the BTB core protein (Lu et al., 2021). NPs can cross the BTB and concentrate in the reproductive organs. NPs can accumulate in the testis, damaging Sertoli cells, Leydig cells, and germ cells and resulting in reproductive organ failure and poor sperm quality (Wang et al., 2018). Microplastics (MPs) have emerged as hazardous materials, and their potential toxicity has attracted widespread attention. Exposure to MPs may result in the breakdown of BTB integrity and spermatogenic cell apoptosis (Li et al., 2021). Microcystin-LR (MC-LR) is a new environmental toxin generated by cyanobacteria that is harmful to both wildlife and humans. MC-LR causes hyperphosphorylation of specific proteins, which activates several signaling pathways that lead to inflammation and the breakdown of the BTB (Zhang S et al., 2021). Polycyclic aromatic hydrocarbons (PAHs) are volatile hydrocarbons produced when coal, petroleum, wood, tobacco, organic macromolecular compounds and other organic compounds are incompletely burned and are important environmental and food pollutants. BaP and Py elicit Sertoli cell apoptosis and BTB disruption involving mitochondrial dysfunction (Zhang et al., 2022). Mechanisms related to environmental pollutants, such as oxidative stress, inflammation, apoptosis, and the breakdown of barrier structures, are now considered to contribute to reproductive toxicity and may cause damage at the molecular and genetic levels. Toxicant-mediated BTB and Sertoli cell dysfunction that leads to male infertility can be therapeutically controlled and perhaps cured if the molecular targets and underlying processes are discovered and understood. This provides hope to many men with unexplained infertility (Zhang L et al., 2021).

In some cases of male infertility, such as varicocele (VC) and cryptorchidism, disruption of the BTB and changes in the expression of its proteins are thought to be one of the mechanisms of spermatogenesis dysfunction. The expression of claudin-11 (Pan et al., 2018), E-cadherin and alpha-catenin (Ha et al., 2011) in a rat model of VC has been shown to be prominently downregulated, leading to the destruction of the integrity of the BTB. Deregulation of Cldn11 in the Sertoli cells of VC testes may be caused by an increase in proinflammatory cytokines, resulting in changes in the permeability of the BTB and immunological obstacles to normal spermatogenesis (Oh et al., 2016). In another study (Zhang L et al., 2021), the structure and function of anchoring junctions (AJs) in the testes of rats were discovered to be disturbed by VC. Loss of normal BTB function and decreased spermatogenesis have been found in undescended testes, suggesting that aberrant CLDN11 organization may induce undescended testes-associated male infertility (Kato et al., 2020). An unhealthy lifestyle, characterized by, for example, consuming a high-fat diet leading to obesity, chronic stress, and lack of sleep, can also affect sperm quality through damage to the BTB. Obesity is a known risk factor for infertility, and obesity-induced chronic inflammation affects spermatogenesis in the male genital tract (Fan et al., 2017). In obese animals, the production of testicular proinflammatory cytokines has been shown to be elevated, the BTB to be disturbed, and sperm mobility to be drastically decreased (Su et al., 2021). The BTB is disturbed by a cholesterol-rich diet that substantially and time-dependently enhances lipid accumulation in the seminiferous tubules. In the seminiferous tubules of rabbits and rats fed a cholesterol-enriched diet, total protein levels of the TJ proteins ZO-1 and occludin are increased, and TJ protein distribution patterns are significantly altered (Morgan et al., 2014; Fan et al., 2015). Psychological stress may have a significant impact on spermatogenesis since suppression of the hypothalamic-pituitary-gonadal axis produces a drop in testosterone levels, which causes alterations in Sertoli cells and the BTB, resulting in spermatogenesis arrest (Morgan et al., 2014; Fan et al., 2015). Chronic stress has been shown to impact critical proteins and sperm characteristics of the BTB, including BTB and ZO-1 integrity as well as CLDN11 levels (Kolbasi et al., 2021). Sleep deprivation reduces the expression of TJ proteins, actin, and androgen receptor as well as sperm viability and motility as a result of blood-testis changes (Dominguez-Salazar et al., 2020).

The direct detection of the BTB in humans has not been accomplished due to technological limitations, but research on its critical protein and gene expression features may be the key to future advances in male infertility. In humans, claudin-11 might be an important component of the BTB. Normal (localization to the basal component of seminiferous tubules) and aberrant (diffuse expression in Sertoli cells/extremely low or no expression) patterns of claudin-11 protein have been identified. In men with defective spermatogenesis, the claudin-11 pattern is strong, but the fraction of localization is changed in Sertoli cell-only syndrome and primary spermatocyte maturation arrest (Stammler et al., 2016). The proportion of men with nonobstructive azoospermia with an aberrant claudin-11 expression pattern, particularly those with Sertoli cell-only syndrome, is substantially higher than that of men with obstructive azoospermia (Chiba et al., 2012). In males with primary seminiferous tubule failure, the spatial arrangement of claudin-11 and connexin-43 is changed (Haverfield et al., 2013). Klinefelter syndrome (KS) is the most frequent hereditary cause of infertility in males; the expression of the BTB proteins connexin-43 and claudin-11 is drastically decreased in KS patients (Giudice et al., 2019). In nonobstructive azoospermia patients, claudin-11, occludin, and ZO-1 are associated with increased apoptosis and unstained/irregular TJ development (Aydin et al., 2020). Sox8, an SRY-related HMG box transcription factor, is required for germ cell development in Sertoli cells by regulating BTB integrity. In Chinese males, harmful mutations in the SOX8 gene may not be a prevalent cause of oligo/azoospermia (Zhang et al., 2020). Intracellular adhesion molecule-1 (ICAM-1) is a component of the BTB and belongs to the immunoglobulin cell adhesion molecule family. The single nucleotide polymorphism ICAM-1 rs5498 (1462A>G) is linked to an increased incidence of obstructive azoospermia, with a considerably greater prevalence of AG heterozygotes among infertile males (Zhang et al., 2020).

### Architecture and dynamics of the blood-testis barrier

The BTB is composed of Sertoli cells and is necessary for the development and maturity of spermatogenic cells. The BTB loses its integrity when the tight link between Sertoli cells is broken, resulting in spermatogenesis problems. Some important key proteins or components are constantly being discovered, such as PTBP1 (Yang et al., 2021), noncollagenase domain (NC1)-peptide (Liu et al., 2021), a 22-amino-acid peptide (22AA) (Zhu et al., 2020), ubiquitously expressed transcript (Thomas et al., 2020), TBC1D20 (Thomas et al., 2020), SENP3 (Wu et al., 2017), BT-IgSF (Pelz et al., 2017), Pin1 (Islam et al., 2017), ANXA2 (Chojnacka et al., 2017), AKAP9 (Venkatesh et al., 2016), GATA4 (Schrade et al., 2016), and intercellular adhesion molecule-1 (Mruk, 2016), that are required for the structural integrity of the BTB. Regulation of BTB dynamics is also an important research area. Mammalian target of rapamycin (mTOR) is a fundamental regulator of the cellular metabolic phenotype that is involved in almost every aspect of cellular function. It integrates actin cytoskeleton organization (Jesus et al., 2017). It regulates BTB dynamics during spermatogenesis through mTORC1 and mTORC2’s “Yin” and “Yang” effects. The level of Rictor/mTORC2 expression is linked to the integrity of the BTB (Mok et al., 2013b). The mTORC1/rpS6 signaling cascade controls BTB dynamics, and treating Sertoli cells with rapamycin inhibits mTORC1 and increases the Sertoli cell barrier, making it “tighter” (Yan et al., 2019). This is one of the key BTB opening and closing processes that has been well documented and tested. Bisphenol S may cause BTB disruption by altering cytoskeletal architecture, which is mediated by an imbalance between mTORC1 and mTORC2 (Wu et al., 2021). Infection with uropathogenic *Escherichia coli* affects the BTB by disrupting the mTORC1-mTORC2 balance (Lu et al., 2021). c-Yes and c-Src also have similar antagonistic effects. Endocytosed integral membrane BTB proteins have been found to be promoted to the pathway of transcytosis and recycling, allowing internalized proteins to be used to effectively assemble new BTB from the disassembling old BTB, whereas c-Src promotes endocytosed Sertoli cell BTB proteins to endosome-mediated protein degradation, allowing the old BTB to degrade (Xiao et al., 2014). Other components found to regulate BTB dynamics are Klf6 (Wang et al., 2019), Cdc42 (Su and Cheng, 2019), CAMSAP2 (Mao et al., 2019), laminin 2 (Gao et al., 2017b), and laminin alpha 2 (Gao et al., 2017a). Sertoli cell TJs and TJ proteins might be regulated by FSH, testosterone, and paracrine hormones as possible mediators of junction formation and disassembly during the translocation process (Stanton, 2016). These biological breakthroughs have resulted in a slew of new technical advancements in the treatment of male infertility, including the repair of damaged BTB in nonobstructive azoospermia (NOA) patients with spermatogenic maturation arrest and hypospermatogenesis (Zhu et al., 2019) and manipulation of TJ proteins to promote stem cell homing efficiency (Kanatsu-Shinohara et al., 2018).

### Blood-testis barrier and immunity

The immunological barrier prevents the immune system from reaching the bulk of autoantigenic germ cells and sequesters them. This, together with the testis’ general immunological privilege, prevents harmful immune responses against autoantigenic germ cells (Mital et al., 2011). The BTB serves to protect luminal germ cells from the circulatory and lymphatic systems, resulting in an immune-privileged milieu for meiosis completion when combined with local immunosuppression. The immune response’s destiny is controlled by cellular components, which can transform the reaction from immunodestructive to immunoprotective, resulting in immunological privilege (Kaur et al., 2013). Male fertility can be hampered by infection and inflammation in the reproductive system. Toll-like receptors (TLRs) as well as inflammatory cytokines and their signaling pathways play critical roles in regulating Sertoli cell activity and responses to reproductive hormones as well as encouraging immunological responses inside the testis. Many of the negative effects of inflammation on spermatogenesis can be attributed to increased production of inflammation-related gene products in the circulation and testis, which disrupt spermatogenic cell development and survival as well as the Sertoli cells’ ability to provide spermatogenesis support (Hedger, 2011). The BTB is important for protecting haploid germ cells from immune assault, and IL6 may have a role in the downregulation of occludin expression and modification of BTB permeability seen in autoimmune orchitis rats (Perez et al., 2012). Th17 cells and their signature cytokine IL17A have been shown to have a role in the development of autoimmune orchitis. IL17A promotes immune cell recruitment to the testicular interstitium while also impairing BTB function (Perez et al., 2014). IL-6 perturbs the integrity of the BTB, which changes the usual location and steady-state levels of BTB integral membrane proteins (Zhang et al., 2014). IL-1 frequently causes actin network and cell junction remodeling, resulting in junction disintegration (Lie et al., 2012). MKP-1 is a key endogenous suppressor of innate immune responses involved in controlling BTB barrier dynamics. Immune privilege in the testis is not permanent since an efficient immune response to transplanted tissue may develop and bacterial/viral infections in the testis can be effectively removed. Spermatogenesis takes place mostly in the testis. Infected people may experience lower sperm quality and infertility as a result of Zika virus, mumps virus, human immunodeficiency virus, and other pathogens. Although these viruses cannot directly cross the BTB, the inflammatory factors they secrete can cause the TJ proteins between supporting cells to degrade, disrupting the structure and function of the BTB and allowing viruses to invade the spermatogenic tubules. The interaction of ZIKV E with F-actin causes the F-actin network to reorganize, jeopardizing BTB integrity (Nie et al., 2021). When Sertoli cells are exposed to inflammatory mediators originating from ZIKV-infected macrophages, the ZO-1 protein is degraded, which is associated with enhanced BTB permeability (Siemann et al., 2017). SARS-CoV-2 may cause proinflammatory cytokine upregulation and BTB junctional protein downregulation, resulting in BTB disruption and spermatogenesis impairment (Peirouvi et al., 2021).

### Blood-testis barrier in oxidative stress and apoptosis

Reactive oxygen species (ROS) may play a role in 30–80% of infertile patients (Takeshima et al., 2021). An imbalance in the physiology of the body between antioxidants and ROS causes oxidative stress. Toxicants in the environment, chemotherapeutic medications, heat, and illness disrupt Sertoli cell structure and function, causing damage to the BTB and a link to excessive ROS generation (Agarwal et al., 2003). These health risks can be ameliorated via oral administration of some bioactive agents, including betaine (Jiang et al., 2019), aucubin (Ma et al., 2019b), salidroside (Jiang et al., 2020), paeonol (Morsy et al., 2020), betulinic acid (Lin et al., 2022), and cordycepin (Huang et al., 2022). Ascorbic acid, a well-known ROS scavenger, may protect against BTB degradation by inhibiting oxidative stress (Chen et al., 2018). Antioxidant drugs such as metformin (Ye et al., 2019), fluvastatin (Gurel et al., 2019), and MitoQ (Zhang et al., 2019) also reduce oxidative stress-induced BTB damage on male fertility. Apoptosis is one of the most well-known quality-control systems in the testis. Increased apoptosis may have negative consequences on sperm production, ultimately jeopardizing male fertility (Zhang et al., 2019). Some antioxidant-rich natural substances, such as morin (Arisha et al., 2019), luteolin, and Lycium barbarum polysaccharide (Hu et al., 2021), also have anti-apoptotic properties, minimizing damage to the BTB. Antioxidant supplementation may be a potentially viable treatment method for both the prevention and relief of BTB damage, according to evidence from these animal investigations.

### Strengths and limitations

To our knowledge, this is the first bibliometric study of trends in BTB research. Through coauthorship, cocitation, and cooccurrence analyses, we built and visualized bibliometric networks using well-known scientometric tools (VOSviewer and CiteSpace). Research on the composition of the BTB and related damage will be of great help in protecting male fertility and developing related contraceptives in the future. We also see that many drugs and components based on the repair of the BTB have great prospects in the treatment of male infertility. Nonetheless, there are certain limitations to our findings. The searches were initially mostly undertaken in the WoSCC. Second, it appears that we only included WoSCC studies in English. Furthermore, because this is a new and evolving research topic, we may have underestimated the contribution of newly published studies to various analyses due to their low citation frequency, although some of the studies were published in high-quality journals. However, we believe that this study may still be used to convey the overall position and general development in this sector.

## Conclusion

According to our findings, China and the United States are important contributors to research on the BTB. Cheng C. Yan of the US Population Council is a pioneering scientist who has made substantial contributions to this subject. The bulk of relevant papers have been published in high-impact journals, implying that advancement in this subject is significant. The keywords indicate that a variety of compositional features (TJs, cytoskeletons, adherens junctions), cell types (Sertoli cells, germ cells, Leydig cells, stem cells), reproductive toxicity (cadmium, NPs, bisphenol-a), and relevant mechanisms (spermatogenesis, apoptosis, oxidative stress, dynamics, inflammation, immune privilege) are areas of interest in BTB research. The research has shifted away from identifying the BTB toward understanding its architecture, activation mechanisms, and male infertility functions.

## Data Availability

The original contributions presented in the study are included in the article/supplementary material, further inquiries can be directed to the corresponding author.
